# Supplementary material for: The interactome of intact mitochondria by cross-linking mass spectrometry provides evidence for coexisting respiratory supercomplexes
Source: Mol Cell Proteomics. 2017 Dec 8;17(2):216–32. doi: 10.1074/mcp.RA117.000470 (PMC5795388; doi:10.1074/mcp.RA117.000470)
Supplement: Supplemental Data [file supp_RA117.000470_133922_0_supp_23978_fzffwf.zip › spectra_annotation/mito_DR_spectra_annotation/497-1-1-1-7-1.pdf]

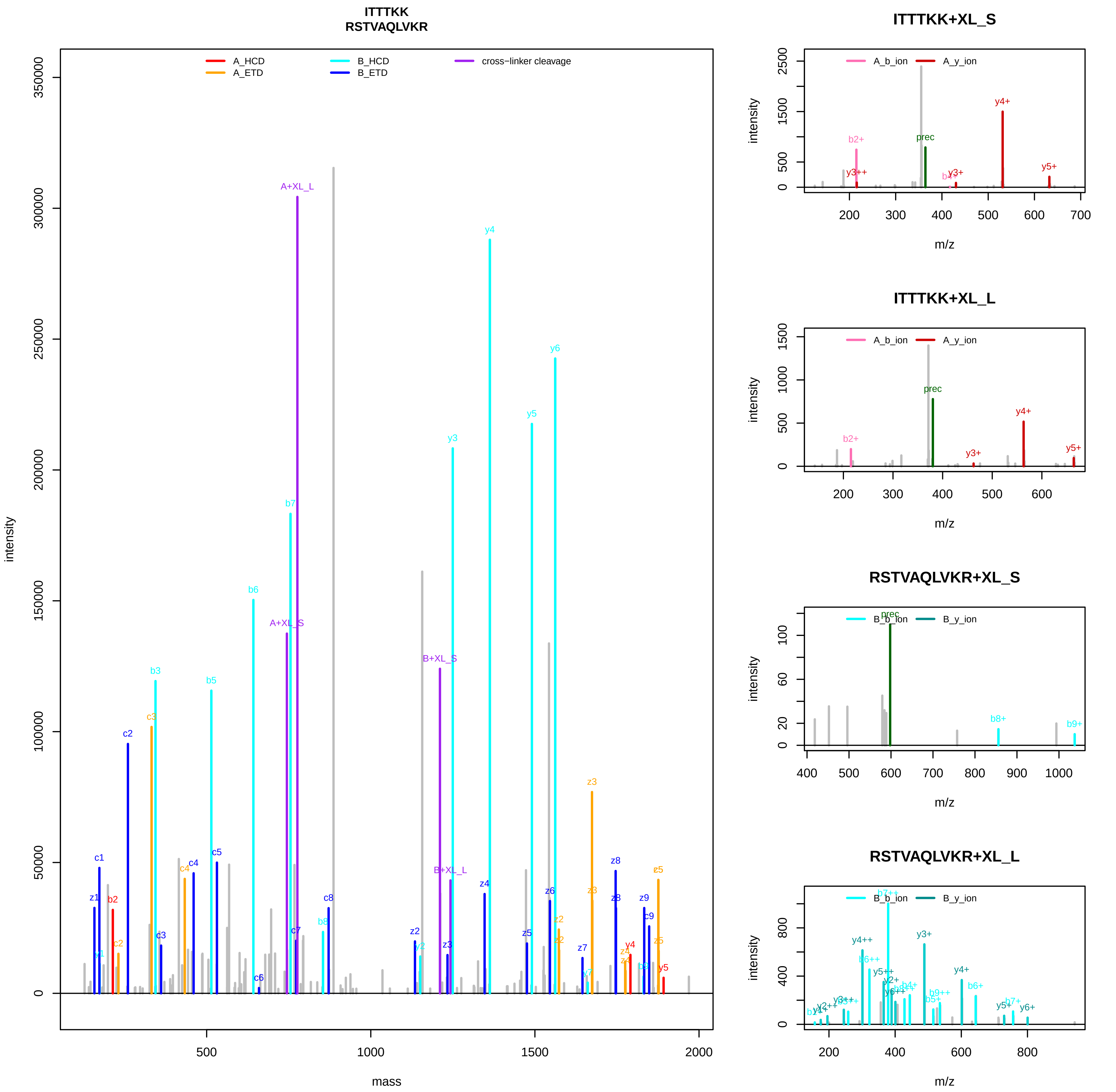
ITTTKK
RSTVAQLVKR
A_HCD
A_ETD
B_HCD
B_ETD
cross−linker cleavage
A+XL_L
A+XL_S
B+XL_S
B+XL_L
y4
y6
y5
y3
b7
b6
b5
b3
c3
c2
c1
z1
b2
c4
c5
c8
c7
c6
b8
z2
y2
z3
z4
z5
z6
z3
z2
z7
y7
z8
z9
c9
y4
z4
z5
y5
intensity
mass
0
50000
100000
150000
200000
250000
300000
350000
500
1000
1500
2000
ITTTKK+XL_S
A_b_ion
A_y_ion
y4+
prec
b2+
y3++
b4+
y3+
y5+
intensity
m/z
ITTTKK+XL_L
RSTVAQLVKR+XL_S
B_b_ion
B_y_ion
b8+
b9+
RSTVAQLVKR+XL_L
b7++
y4++
y3+
b6++
y5++
y2+
y6++
b8++
b4+
b9++
y4+
b6+
b5+
b7+
y5+
y6+
